# Temporomandibular Disorders, Bruxism, Perceived Stress, and Coping Strategies among Medical University Students in Times of Social Isolation during Outbreak of COVID-19 Pandemic

**DOI:** 10.3390/healthcare10040740

**Published:** 2022-04-15

**Authors:** Klara Saczuk, Barbara Lapinska, Adam Wawrzynkiewicz, Alicja Witkowska, Heber Isac Arbildo-Vega, Monika Domarecka, Monika Lukomska-Szymanska

**Affiliations:** 1 Department of General Dentistry, Medical University of Lodz, 92-213 Lodz, Poland; klara.saczuk@umed.lodz.pl (K.S.); barbara.lapinska@umed.lodz.pl (B.L.); monika.domarecka@umed.lodz.pl (M.D.); 2 Department of Clinical Chemistry and Biochemistry, Medical University of Lodz, 90-419 Lodz, Poland; adam.wawrzynkiewicz@stud.umed.lodz.pl; 3 Private Dental Practice “Ecodentica”, 80-126 Gdansk, Poland; witkowska.alka@gmail.com; 4 Department of General Dentistry, Dentistry School, Universidad San Martín de Porres, Chiclayo 14012, Peru; harbildov@usmp.pe; 5 Department of Human Medicine, Human Medicine School, Universidad San Martín de Porres, Chiclayo 14012, Peru; 6 Department of General Dentistry, Dentistry School, Universidad Particular de Chiclayo, Chiclayo 14012, Peru; 7 Department of Stomatology, School of Stomatology, Universidad Alas Peruanas, Lima 15072, Peru

**Keywords:** temporomandibular disorders, bruxism, stress, COVID-19, perceived stress, coping strategies, medical students

## Abstract

The COVID-19 pandemic caught universities along with their students off-guard, enforcing online education. Fear of the unknown, disinformation, and isolation resulted in an increased stress level in the entire population. Medical university students are particularly endangered with high stress levels and developing TMD. Temporomandibular disorders (TMD) are of multifactorial etiology, and manifest with jaw dysfunction, masticatory muscle tension or pain, as well as headache. Though bruxism can act as an exacerbating factor for TMD, stress can also play crucial role in the onset. The study aimed to measure occurrence of TMD and bruxism symptoms in the medical student population, asses the stress level, and evaluate adopted stress-coping strategies during the COVID-19 pandemic outbreak. A survey study was performed among 1018 students at Medical University of Lodz during April 2020. A self-designed questionnaire for screening TMD and bruxism symptoms, Perceived Stress Scale (PSS-10), and Brief-COPE questionnaires were applied. TMD and bruxism symptoms were observed in the majority of subjects during social isolation. The perceived stress levels were significantly higher in those experiencing TMD and bruxism symptoms. Mostly maladaptive, emotion-focused coping strategies were chosen by study subjects experiencing high levels of stress. Choosing Self-Blaming as a coping strategy is the strongest predictor of perceived stress.

## 1. Introduction

In the beginning of 2020, the epidemic state and implemented precautions forced universities worldwide to close down and begin online teaching [1,2]. Poland was no exception, which resulted in suspending stationary classes in Polish higher education. Starting from 12 March 2020, all university classes in Poland have been moved to online mode. Some of the classes were conducted via e-learning, and others in real-time via different online platforms, e.g., MS Teams, Zoom, and Skype, allowing for video conference mode, yet it was not mandatory for the students to use this mode during classes. Hence, students had no direct face-to-face contact with each other, apart from those who were already roommates. Moreover, further directives of university government instructed students living in dormitories to return to their homes so that the buildings could be used as temporary hospitals for patients suffering from COVID-19. Surprisingly, homecoming could have helped junior year students to find support and comfort from the family members at home.

Moreover, places such as restaurants, bars, clubs, and cinemas were also closed to avoid any social gatherings. Such enforced isolation of students might have possibly influenced their behavior, and social and educational performance [3]. Some studies have stated that real-life interactions promote cooperation and cooperative behaviors, whereas others have stated that online courses are equally or sometimes more effective than face-to-face classes [4,5].

Around 10–15% of the general population is affected by temporomandibular disorders (TMD) [6]. TMD is a collective term involving disorders of both the temporomandibular joint and masticatory muscles. TMD symptoms may involve pain in the temporomandibular region or in the masticatory muscles; pain radiating behind the eyes, in the face, shoulder, neck, and/or the back; headaches; ear ache or tinnitus; jaw clicking, locking, or deviation; limited jaw opening; clenching or grinding of the teeth; dizziness; and sensitivity of the teeth, lacking oral disease [7].

Bruxism can be an exacerbating factor in TMDs. It affects between 8–31% of the adult population, irrespective of gender [8]. Its severity appears to decrease with age [9]. The International Classification of Sleep Disorders defines bruxism as a “repetitive jaw-muscle activity characterized by clenching or grinding of the teeth and/or by bracing or thrusting of the mandible” [10]. Bruxism is distinguished into two separate forms—awake bruxism (AB) and sleep bruxism (SB) [11]. Awake bruxism is defined as a “masticatory muscle activity during wakefulness that is characterized by repetitive or sustained tooth contact and/or by bracing or thrusting of the mandible”, whereas sleep bruxism as a “masticatory muscle activity during sleep that is characterized as rhythmic (phasic) or non-rhythmic (tonic)” [11]. It was pointed out that neither form of bruxism is a movement disorder or a sleep disorder in otherwise healthy individuals [11].

Leading theory describing the complex etiology of bruxism pertains to an increased impulsivity from the central nervous system [12,13,14]. Theories relating bruxism only to occlusal discrepancies have not gained sufficient clinical support to be considered an important aspect in bruxism etiology [15]. However, various other factors also play a part in the occurrence of bruxism. Those factors can be divided into biological (e.g., neurotransmitter imbalance, co-occurring disorders), genetic (gene polymorphisms), psychological (e.g., stress, coping strategies, personality aspects, social isolation), and external (e.g., nicotine, tobacco, alcohol) [16,17,18]. Among psychological factors, social isolation has shown to have an important role in both mental and physical health [19,20].

Individuals may develop with different responses to stressors. The literature describes various stress-coping strategies [21], and differentiates them on problem-focused strategies (i.e., Planning, Active Coping, Seeking Instrumental Support, and Positive Reframing), emotion-focused strategies (i.e., Self-Blaming, Seeking Emotional Support, Acceptance, Venting, Religion, Humor), and avoidant coping (i.e., Self-distraction, Substance Use, Behavioral Disengagement, Denial) [22]. Some studies found that the most commonly chosen stress-coping strategies in healthy, well-educated, wealthy older populations are Planning and Active Coping [23,24]. Moreover, it was noted that choosing these coping strategies coincide with low perceived stress [24].

It was reported that perceived stress is higher among women than men [25,26,27,28]. Moreover, highest perceived stress was noted among younger, single (unmarried and divorced), unemployed, and less-educated participants [25,28]. As for university students, medical or health science university students in particular, abundant literature reports that in general, they obtain higher perceived stress scores than their gender-matched peers in the general population [29,30,31,32].

In light of these findings, it would be interesting to investigate how social isolation driven by COVID-19 pandemic restrictions can influence stress levels and coping strategies among university students. Therefore, this paper aims to evaluate the relationship between the occurrence of bruxism, perceived stress levels, and coping strategies among the students of the Medical University of Lodz in the time of social isolation caused by the SARS-CoV-2 pandemic.

## 2. Materials and Methods

### 2.1. Study Design

The study was performed from 4 April to 26 April of 2020 among the students at the Medical University of Lodz, of all faculties (i.e., Faculty of Medicine, including Faculty of Dental Medicine with Dental Technology, and Faculty of Biomedical Sciences; Faculty of Pharmacy, including Laboratory Medicine and Cosmetology; Faculty of Health Sciences, including Public Health, Dietetics, Emergency Medicine, Nursing, and Midwifery). All classes were moved to online platforms on 12 March; therefore, during the time of this study, the students had been in isolation for 19–38 days. Out of 8000 students of all faculties of all study years, 1018 participated in the study. The study was blinded/anonymous. The Research Ethics Committee of the Medical University of Lodz approved the investigation (RNN/117/20/KE), and it was conducted according to the principles expressed in the Helsinki Declaration. The students received written information on the purpose and procedures of the study, and they gave written informed consent.

The study consisted of three self-reporting questionnaires: A questionnaire for screening TMD symptoms and possible bruxism, Perceived Stress Scale (PSS-10), and Brief-COPE scale. The questionnaires, transcribed into a Google Forms and uploaded to a free survey platform, were distributed amongst students through students’ mailing lists, the university intranet platform, and student groups on social media (Facebook and MS Teams platform). This method of distribution was agreed upon with the Medical University officials and the Office for Administrative Management of Studies. Students filled the questionnaires online.

**Figure A1:**
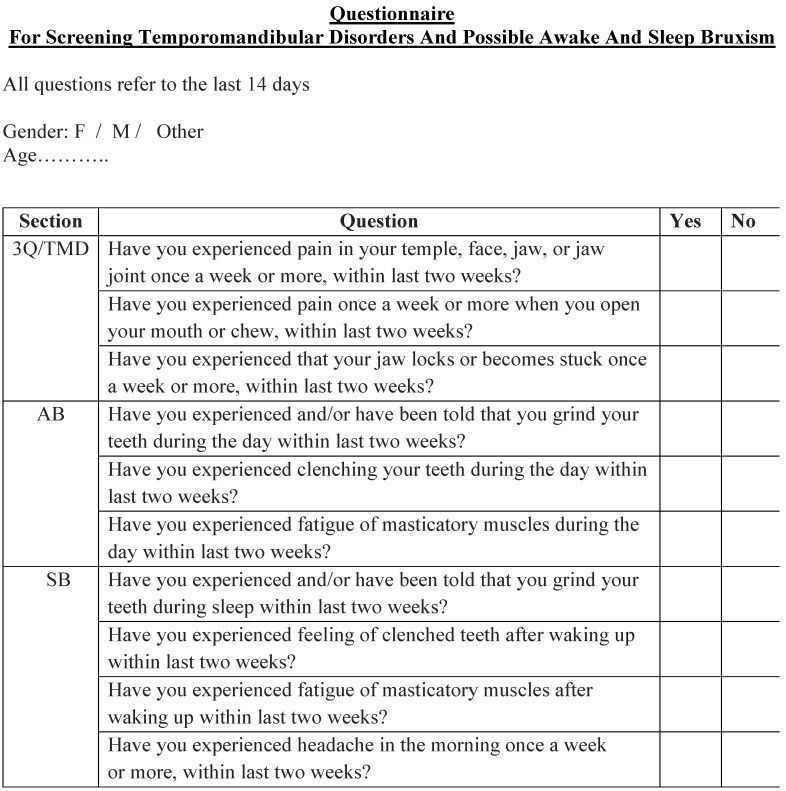
The questionnaire used in the study for screening subjects for TMD symptoms, possible ABW, and possible SB.

For screening TMD symptoms and possible bruxism, the authors used a self-designed, self-report tool matched specifically for symptoms of TMD and bruxism appearing currently (within the last two weeks) (Appendix A, Figure A1). The questionnaire consisted of ten Yes/No questions, grouped in three sections. The first section was based on the 3Q/TMD questionnaire [33,34], whereas the two other sections included questions assessing possible awake and sleep bruxism, respectively. The questions were pertaining to the occurrence of pain in the temple, face, jaw, or jaw joint; pain when opening the mouth or chewing and the jaw locking or becoming stuck (for screening TMD); grinding or clenching teeth during the day or fatigue of masticatory muscles during the day (for possible AB); grinding teeth during sleep, or feeling of clenched teeth after waking up, or fatigue of masticatory muscle after waking up, or headache in the morning (for possible SB). Positive answers to one of the questions indicate possible TMD, AB, and SB, respectively. The 3Q/TMD questionnaire, as well as self-report questionnaires, are acceptable tools for screening TMD symptoms [33,34,35], and to asses possible awake or sleep bruxism [9,11,35,36], respectively.

For stress levels, the PSS-10 questionnaire was used. The PSS-10 is a self-reporting tool comprising 10 statements regarding “how unpredictable, uncontrollable, and overloaded respondents find their lives within the last month”. Each statement on the PSS-10 ranges from 0 (never) to 4 (very often) on a 5-point Likert scale. The PSS-10 involves 6 positive statements (1, 2, 3, 6, 9, and 10), and 4 reverted statements (4, 5, 7 and 8). During the assessment, points from 0 to 4 are awarded for each statement, with reverted statements being re-calculated, and the scores determined for each subject. Total scores range from 0 to 40, and the higher the score, the higher the perceived stress level [37].

Mean PSS-10 scores for TMD symptoms or coping strategies were calculated by adding the PSS-10 scores of subjects experiencing a particular symptom or using a particular coping strategy and dividing it by the number of subjects.

Since all participants were of Polish nationality, the PSS-10 results were converted to the sten scale (standard ten) [38]. The sten scale is the normalized psychological evaluation scale in such a way that the population average is 5.5 sten, and the standard deviation is 2. PSS-10 levels ranging from 0–13 (1–4 sten) were therefore considered low perceived stress, 14–19 (5–6 sten) were considered moderate perceived stress, and high perceived stress was considered to be 20–40 (7–10 sten) [38].

For measuring the most frequently, currently-used coping strategies, the authors used Brief-COPE. The Brief-COPE scale is also a self-reporting tool comprising 28 statements regarding 14 strategies of coping with stress [39]. Each of the 14 strategies (Active Coping, Planning, Positive Reframing, Acceptance, Humor, Seeking Emotional Support, Seeking Instrumental Support, Self-Distraction, Denial, Venting, Substance Use, Behavioral Disengagement, Self-Blaming) corresponds with two particular statements of the 28 in the Brief-COPE [24,39]. All Brief-COPE statements range from 0 (almost never) to 3 (almost always). For each coping strategy, scores are determined during the test by adding the points from two related statements together and then dividing them by two. For each coping strategy, total scores range from 0 to 3, and the higher the score for a specific strategy, the more it is used by the respondent.

### 2.2. Statistical Analysis

For qualitative variables, the structure indices were calculated and expressed in percentage. For measurable variables, test value (z), minimum (min) and maximum (max) values were given and the following characteristics: arithmetic mean (x) and median (Me) as average values, and standard deviation (SD) and the coefficient of variation (CV) as measures of dispersion were calculated. The normality of the quantitative variable distributions was checked using the Shapiro–Wilk test prior to comparison of the averages. As the distributions of variables significantly differed from the normal distribution, the non-parametric Mann–Whitney test was used to compare means in two groups (study and control). The rank correlation coefficient was calculated in the study of the relationship between measurable variables. A level of *p* < 0.05 was considered statistically significant. If theoretical numbers were less than 5, then the Yates amendment was considered, and if smaller than 3, Fisher’s exact test was used, and there was no chi-squared, only *p*.

## 3. Results

The survey was filled by 1018 participants, aged 18–30 years, with the mean age of 21.7 ± 2.5 years. In terms of gender, 790 participants (77.6%) identified as female, 219 (21.5%) as male, and 9 (0.9%) participants identified with another gender. Out of 1018 students that participated in the study, the majority reported the presence of TMD and/or bruxism symptoms during the isolation period.

The study found that TMD and SB symptoms were reported by majority of respondents (77.3% and 58.9%, respectively), whereas AB symptoms were reported by nearly half of them (47.8%). As for possible AB, SB, and TMD symptoms, they were statistically more frequent among women and respondents identifying as another gender than among men (Table 1).

As for PSS-10 results, the median score for the tested population was 22.95 ± 7.28. For the whole group of subjects experiencing TMD/bruxism symptoms, the median PSS-10 was 23.47 ± 7.15, whereas for the whole group of subjects not experiencing TMD/bruxism symptoms, the score was lower and estimated at 19.47 ± 7.23. Yet, these differences were not statistically significant.

In subjects with TMD, and awake and sleep bruxism symptoms, high score ranges of PSS-10 were observed significantly more often in comparison to those without these conditions (Table 2), whereas subjects without possible TMD/AB/SB presented with low PSS-10 score ranges significantly more often.

**Figure 1:**
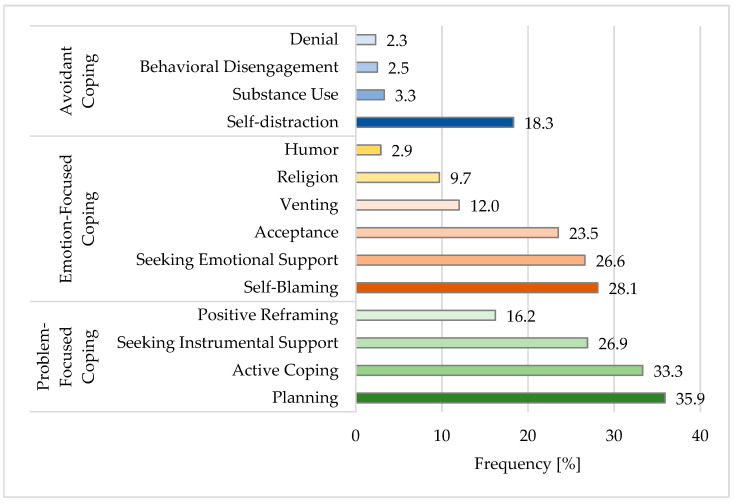
Frequency of choosing Coping Strategies (Brief-COPE) in the tested population.

Planning, followed by Active Coping and Self-Blaming were the most frequently chosen stress-coping strategies among all subjects (Figure 1). Seeking Instrumental and Emotional Support and Acceptance were other coping strategies chosen by more than 20% of subjects.

**Figure 2:**
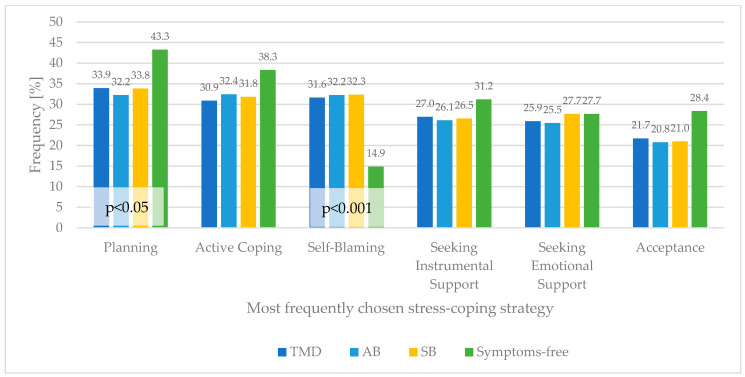
Frequency (percentage of subjects) of choosing most chosen coping strategies among subjects presenting TMD symptoms, possible AB and SB, and symptom-free.

Detailed analysis of most frequently chosen stress-coping strategies by subjects presenting with conditions (possible TMD, awake and sleep bruxism) and symptom-free subjects is given in Figure 2. Self-Blaming was the significantly more often chosen strategy among subjects with TMD symptoms, or possible awake and sleep bruxism than among asymptomatic subjects. Moreover, subjects with TMD symptoms and possible bruxism chose Planning significantly less often than those without these symptoms.

**Figure 3:**
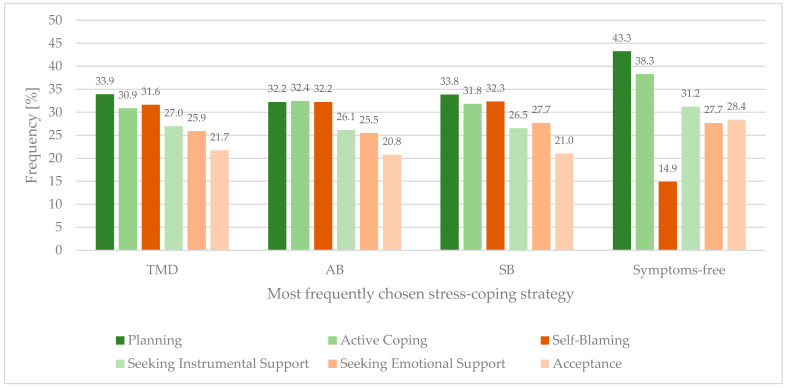
Frequency (percentage of subjects) of choosing coping strategies among subjects presenting TMD symptoms, possible AB and SB.

When analyzing groups of subjects (Figure 3) presenting TMD symptoms and those with possible AB, it turned out that among top six frequently chosen stress-coping strategies, Acceptance followed by Seeking Emotional Support and Seeking Instrumental Support were chosen significantly less often than Planning, Active Coping, or Self-Blaming (Table A1 and Table A2). In the case of subjects with possible SB, Acceptance followed by Seeking Instrumental Support were chosen significantly less often than Planning, Active Coping, or Self-Blaming (Table A3).

**Figure 4:**
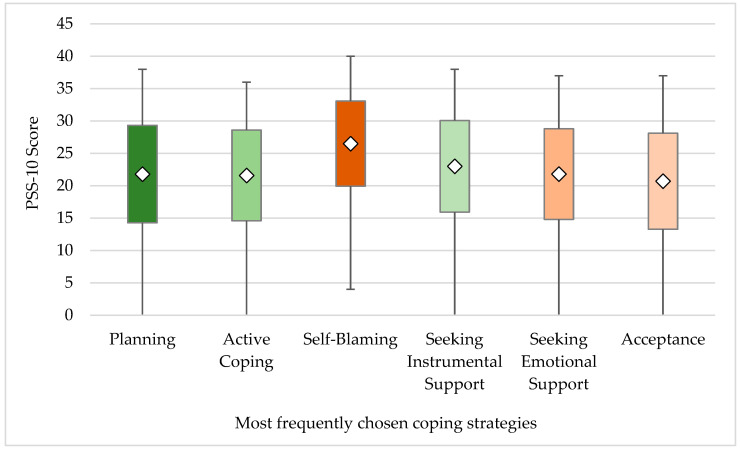
PSS-10 result vs. most frequently chosen coping strategies among subjects in the tested population.

When contrasting the PSS-10 score with the most frequently chosen stress-coping strategies (Figure 4), it turned out that subjects who chose Self-Blaming as coping strategy obtained the highest mean PSS-10 score (26.5 ± 6.58), followed by the subjects who chose Seeking for Instrumental Support (23.0 ± 7.06). Next, those who chose Planning, Seeking for Emotional Support, and Active Coping strategies scored a similar mean PSS-10 result: 21.8 ± 7.53; 21.8 ± 7.0 and 21.6 ± 7.01, respectively. The lowest mean PSS-10 was obtained by subjects who chose Acceptance (20.7 ± 7.41) as the most common stress management strategy.

A correlation (*p* < 0.05) was found between PSS-10 score and the frequency of choosing a specific stress-coping strategy for most of the reported strategies, aside from Religion and Seeking Instrumental Support (*p* > 0.05) (Table 3). The higher the PSS-10 score, the less frequently problem-focused coping strategies, such as Planning, Active Coping, Seeking Instrumental Support, and Positive Reframing, or emotion-focused strategies, i.e., Acceptance, Humor, Religion, and Seeking Emotional Support, were chosen (negative correlation).

On the contrary, the frequency of choosing emotion-focused coping, such as Self-Blaming and Venting, or avoidant coping, i.e., Self-Distraction, Substance Use, and Behavioral Disengagement or Denial, rises together with a high PSS-10 score (positive correlation).

All correlations are found to be weak except for the Self-Blaming strategy, having moderate positive correlation with the PSS-10 score.

## 4. Discussion

Social isolation, as imposed by the COVID-19 pandemic, was predicted to have an impact on mental health [40]. Families experiencing quarantine and/or lockdown measures together reported feeling a loss of freedom of movement, along with a loss of community networks [41]. However, a different study showed that even though the pandemic amplified the feelings of feeling terrified, helpless, and apprehensive, it also increased caring for family members’ feelings among 64.7% of the respondents [42]. Still, it has been stated that the negative consequences for both psychological and emotional wellbeing could be of similar significance as the physical ones [43]. Research shows that levels of stress and anxiety have increased both due to the pandemic itself and social isolation [44].

For screening TMD and bruxism symptoms, the study used a self-designed, self-report tool, created based on 3Q/TMD questionnaire [33], with additional questions pertaining to possible awake and sleep bruxism. In the present study, 77.3% students of Medical University observed TMD symptoms in themselves during the pandemic outbreak. Despite the fact that using the questionnaires could pose some bias, and self-reported TMD/bruxism symptoms could be exaggerated, most of the recent studies screening TMD symptoms among studied populations during the COVID-19 pandemic outbreak mainly used questionnaires [35,45,46,47]. There is no available pre-pandemic data on screening TMD symptoms and/or possible bruxism in the tested university population; however, another study, performed a few years before the COVID-19 pandemic outbreak, using research diagnostic criteria for temporomandibular disorders (RDC/TMD), reported that 54% of Polish students present TMD symptoms [48].

Among this study’s subjects, women, as well as subjects identifying with another gender, significantly more often suffered from symptoms indicating possible AB, SB, and TMD. This is consistent with previous findings indicating female gender to be a predictor for possible AB, SB, and TMD [35,48].

Other studies have also shown that stress could be an important factor in developing TMD, both in the general population and in students [49,50,51]. Moreover, the study discovered that more than 30% of subjects reported symptoms of possible AB, such as a feeling of clenched teeth, and fatigue of masticatory muscles during the day. Interestingly, in the present study, nearly one quarter of subjects reported sleep grinding. Another study conducted on the Polish population during the COVID-19 outbreak showed an aggravation of TMD symptoms, as well as awake and sleep bruxism in subjects while in lockdown [35]. Furthermore, studies show that the COVID-19 pandemic has caused notable and detrimental effects on the psycho-emotional status of both Israeli and Polish populations, which resulted in an increase of their bruxism and TMD symptoms [35,52]. Moreover, the duration of isolation could also be a factor in self-perceived TMD. A study conducted in Italy during the last part of the COVID-19 lockdown (18 April–3 May) showed that 60.8% of subjects reported that TMD symptoms started during the last 90 days, whereas 51.4% claimed that their symptoms worsened during the last 30 days [53].

In the present study, to evaluate the stress levels of the subjects, the PSS-10 was implemented. The PSS-10 questionnaire was chosen from among three existing PSS versions to evaluate the level of perceived stress. PSS-10 was considered the most reliable, valid, and, of psychometric properties, superior to those of both PSS-14 and PSS-4 [54]. The higher the PSS-10 score, the higher the level of perceived stress. The median PSS-10 score for the tested population at the pandemic outbreak was 22.95 ± 7.28. It should be noted that studying in Medical University in general is regarded as emotionally challenging, and the students more often suffer from anxiety or depression [55,56]. The studies performed during pre-pandemic times on the population of Polish students from medical and health science disciplines showed that the median score of the PSS-10 ranges from 18.6 ± 6.95 up to 22.78 ± 3.87 [28,57,58,59]. In comparison to those results, it seems that the perceived stress at the pandemic outbreak measured in this study was only a bit higher. However, the majority of the respondents in this study presented with high levels of perceived stress (PSS-10 score higher than 20). This is consistent with the result of another survey held during the outbreak of COVID-19 reporting 30% of participants with high PSS-10 levels [60].

An interesting finding of this study is that all subjects experiencing symptoms of TMD and bruxism achieved a statistically significantly higher mean PSS-10 score than subjects without bruxism symptoms. This result corresponds with research validating a relationship between stress and the occurrence of bruxism [24,61]. Furthermore, the relationship between bruxism and stress is confirmed by studies showing elevated levels of urinary catecholamines in people suffering from bruxism [62,63]. Moreover, the presence of anxiety and its positive correlation with bruxism have also been confirmed [64]. However, in light of other studies questioning the correlation between stress and bruxism, this topic still requires further research [65,66,67].

All TMD-symptomatic groups presented with higher mean scores of PSS-10 than the asymptomatic groups. There are several possible explanations for the interpretation of this finding. TMD and awake bruxism symptoms are known to occur during high focus or performing mental tasks [68]. Therefore, the above-mentioned symptoms could be the result of high mental engagement required during newly introduced online classes. Moreover, it should be noted that individuals exhibit considerable variability in their perception and adaptation to aversive and stressful stimuli [69]. Therefore, even low levels of stress could cause the occurrence of bruxism or TMD symptoms in some individuals and vice versa.

The most surprising results of this study were those in terms of coping strategies. The authors used the Polish-adapted version of the Brief-COPE questionnaire. It was chosen due to its confirmed premise that an individual’s preferred coping strategies remain comparatively unchanged throughout different stressors [70]. Coping behaviors can be categorized as problem-focused, emotion-focused, or avoidant [22]. The present study showed that in general, students most often chose problem-focused coping strategies, such as Planning (35.9%), Active Coping (33.3%), or Seeking Instrumental Support (26.9%), which involve dealing with the stressor by adapting the stressor. The adaptive coping was reported to be linked to positive psychological and physical health under stressful circumstances [71].

Still, some of the subjects chose Self-Blaming (28.1%), Seeking Emotional Support (26.6%), or Acceptance (23.5%), which are regarded as emotion-focused coping strategies. Maladaptive coping is a risk factor for negative psychological and physical health [72]. These findings are in line with surveys conducted among university students concerning students’ preferred ways of coping with pandemic-induced stress. One study showed that students’ preferred way of coping was seeking social support, whereas the other stated that it was “following strict personal protective measures and reading up about COVID-19, its prevention and ways of transmission”, which could be constituted as Planning or Active Coping. Unfortunately, failing to develop such positive, problem-focused coping strategies may seriously affect students’ academic ability both during the COVID-19 pandemic and in general [73,74]. Another study conducted among Polish students during the COVID-19 pandemic outbreak found that the dominant coping strategies they chose were emotion-focused strategies, i.e., Acceptance, Seeking Emotional Support, and Planning. The least frequently chosen strategies were: Substance Use, Denial, Behavioral Disengagement, and Religious Coping [75]. The present study found that choosing coping strategies, such as Planning, Active Coping, Acceptance, and Seeking Instrumental or Emotional Support, is related to low levels of perceived stress (low PSS-10 score). Even though students showed various strategies of coping, it is advised to address students’ mental health during the COVID-19 pandemic [74].

Another highly interesting finding of this study is the difference between sleep and awake grinders in the most often chosen coping strategies. Both groups of subjects most often chose adaptive, problem-focused coping, such as Planning and Active Coping. However, whereas sleep grinders chose to Seek Emotional Support, awake grinders often chose Self-Blame as a coping strategy. This finding complies with research suggesting that sleep bruxers more often seek emotional/social support [76]. At the same time, it is in contrast with research stating that sleep bruxers tend to use more maladaptive coping strategies, such as Escape, Rumination, Self-Blame, Resignation, or Avoidance [77,78]. This fact suggests that the link between both forms of bruxism and coping strategies is complex and needs to be further investigated. All in all, the present study found that subjects using the Self-Blame coping strategy achieved the highest PSS-10 scores. Our finding is replicated by recent literature, as it was reported that choosing Self-Blaming is the strongest predictor of perceived stress [79]. In our study, Self-Blaming was the significantly more often chosen stress-coping strategy among subjects suffering from TMD symptoms and possible awake and sleep bruxism. Respondents presenting with these conditions noted high ranges of PSS-10 scores significantly more often.

In study subjects choosing Planning and Active Coping, the calculated PSS-10 score was slightly above the lower range value for high perceived stress. These facts would be in agreement with research suggesting that bruxers, and especially awake bruxers, could show adaptive coping strategies while still showing higher scores in stress and anxiety [80]. This finding might suggest that even though an individual might display positively-regarded coping strategies, it still does not protect them from the occurrence of bruxism symptoms.

This could mean that stress levels play a more significant role than coping strategies in developing TMJ disorders. The present study also found weak negative correlation between choosing an adaptive, problem-focused coping strategy and the obtained PSS-10 score, meaning the higher frequency of choosing, e.g., Planning, Active Coping, or Positive Reframing, the lower the PSS-10 result. More detailed analysis of the results showed that subjects feeling fatigue of masticatory muscles and clenched teeth during the day, headache after waking up, and problems opening the mouth in the morning obtained low PSS-10 scores significantly more often than the asymptomatic group. It seems that the relationship between stress-related coping strategies and TMJ is complex and requires further research [81].

All subjects showing bruxism symptoms chose Planning, Active Coping, and Self-Blaming as their top three coping strategies. This could be explained by research proving that even though the anxiety about the current pandemic is linked with an increase of stress and negative emotion, the preferred coping strategies remain unchanged [24,57,82]. Therefore, despite experiencing symptoms of TMD, the respondents could have had problem-oriented and active coping strategies before the onset of the pandemic. Moreover, those same authors state that problem-oriented and active coping strategies are only weakly associated with lower anxiety. Other studies show that patients with bruxism tend to utilize maladaptive coping strategies, such as Self-Blaming and Self-Distraction [24,76]. However, those same sources did not present problem-oriented coping strategies among bruxers. In fact, Planning was statistically significantly more often chosen by non-bruxers than bruxers [24].

The results of this study must be considered carefully, as there are a few limitations to report. The first is the short observation period—during the survey, the students had been in social isolation for approximately 15–45 days, which could result in impaired data. The second was the lack of pre-pandemic results from the same sample for proper comparison. The comparison of obtained results with pre-pandemic data would add greatly to the value of the study. Regretfully, similar to another study reporting on TMD and/or bruxism symptoms developing during the pandemic outbreak [35], the current study did not present pre-pandemic data retrieved from the tested population. Yet, another study on patients already affected by TMD symptoms before lockdown and questioned during it, showed an increase of parafunctions and sleep disorders [47]. Another aspect not considered in the present study is the aggravation of observed symptoms, as well as subjects’ pre-existing mental health disorders. Recent research shows that people with mood or anxiety disorders were more negatively affected by COVID-19 compared to those without them [83]. Moreover, another study [45,46] carried out among physiotherapy students observed that depression, as well as distressed personality, may contribute to the development of TMD symptoms. Furthermore, the study did not take subjects’ chewing habits and sleeping position into account, which are both said to have a possible influence on experiencing TMD symptoms [84,85,86,87]. Moreover, the authors decided to use a self-report TMD questionnaire due to mandatory social isolation for students. However, though careful clinical examination is crucial, the 3Q/TMD questionnaire was chosen specifically for its reproducibility and validity [34]. Furthermore, early diagnosis with the use of screening tools is said to direct the practitioner to more precise diagnostic procedures among the large range of differential diagnostic techniques [88]. In secondary care, screening techniques can help determine which differential diagnostic procedures should be undertaken to assess the entire scope of a patient’s complaints.

Another limitation of the present study is that the survey was conducted only in students from one Medical University, and it did not consider the year of study of the subjects, nor did it differentiate the results among faculties. A survey conducted in 2020 at another Dental University in Poland showed that the highest level of stress was found among first-year dental students [28]. The authors of the study attributed that result to the high level of competitiveness among freshmen, as well as to the rigorous educational challenges they face entering academia.

Lastly, it has to be underlined that further studies on the long-term effects of the COVID-19 pandemic on the aggravation of TMD and bruxism symptoms among medical students are needed. Regretfully, recent military conflict in the neighboring country of Poland, and tensions related to it, could possibly influence the results of the studies, making them difficult to interpret.

## 5. Conclusions

Within the limitations of the recent study, it can be concluded that in the majority of subjects, TMD/bruxism symptoms were observed during social isolation. The perceived stress levels were significantly higher in those experiencing bruxism symptoms. Mostly maladaptive, emotion-focused coping strategies were chosen by study subjects experiencing high levels of stress. Choosing Self-Blaming as a coping strategy is the strongest predictor of perceived stress. Further studies, which would require large samples and more prolonged time periods, are needed to confirm the link between choosing stress-coping strategies and TMD/bruxism symptoms.

## Figures and Tables

**Table 1:** Frequency of TMD symptoms and possible AB and SB among genders in the tested population.

**TMD** **Symptoms**	**Gender**						**Total**	
	**Female**		**Male**		**Other**			
	n	%	n	%	n	%	n	%
Yes	641	81.1	138	63.0	8	88.9	787	77.3
No	149	18.9	81	37.0	1	11.1	231	22.7
Sum	790	100.0	219	100.0	9	100.0	1018	100.0
Comparison	chi^2^ = 32.806; *p* < 0.001							
**Possible** **AB**	**Gender**						**Total**	
	**Female**		**Male**		**Other**			
	**n**	**%**	**n**	**%**	**n**	**%**	**n**	**%**
Yes	401	50.8	79	36.1	7	77.8	487	47.8
No	389	49.2	140	63.9	2	22.2	531	52.2
Sum	790	100.0	219	100.0	9	100.0	1018	100.0
Comparison	chi^2^ = 18.083; *p* < 0.001							
	**Gender**						**Total**	
**Possible** **SB**	**Female**		**Male**		**Other**			
	**n**	**%**	**n**	**%**	**n**	**%**	**n**	**%**
Yes	482	61.0	113	51.6	5	55.6	600	58.9
No	308	39.0	106	48.4	4	44.4	418	41.1
Sum	790	100.0	219	100.0	9	100.0	1018	100.0
Comparison	chi^2^ = 6.323; *p* < 0.05							

**Table 2:** The PSS-10 score ranges in subjects in the tested population.

**PSS-10 Score Range**	**TMD Symptoms**				
	**Yes**		**No**		**Total**
	**n**	**%**	**n**	**%**	**n**
Low	65	8.3	52	22.5	117
Moderate	134	17.0	59	25.5	193
High	588	74.7	120	52.0	708
Total	787	100.0	231	100.0	1018
Comparison	chi^2^ = 51.697; *p* = 0.0000				
**PSS-10 Score Range**	**Possible Awake Bruxism**				
	**Yes**		**No**		**Total**
	**n**	**%**	**n**	**%**	**n**
Low	35	7.2	82	15.4	117
Moderate	86	17.7	107	20.2	193
High	366	75.1	342	64.4	708
Total	487	100.0	531	100.0	1018
Comparison	chi^2^ = 20.115; *p* = 0.0000				
**PSS-10 Score Range**	**Possible Sleep Bruxism**				
	**Yes**		**No**		**Total**
	**n**	**%**	**n**	**%**	**n**
Low	45	7.5	72	17.2	117
Moderate	95	15.8	98	23.5	193
High	460	76.7	248	59.3	708
Total	600	100.0	418	100.0	1018
Comparison	chi^2^ = 38.448; *p* = 0.0000				

**Table 3:** Correlation of PSS-10 result vs. most frequently chosen coping strategies.

PSS-10 Score versus Coping Strategy	Rank Correlation Coefficient	*t* Test	Significance *p*
Planning	−0.140	−4.500	0.0000
Active coping	−0.208	−6.784	0.0000
Self-Blaming	0.375	12.887	0.0000
Seeking Instrumental Support	−0.099	−3.164	0.0016
Seeking Emotional Support	−0.015	−0.470	0.6382
Acceptance	−0.192	−6.232	0.0000
Positive reframing	−0.264	−8.718	0.0000
Venting	0.270	8.937	0.0000
Religion	−0.035	−1.108	0.2681
Humor	−0.142	−4.557	0.0000
Self-Distraction	0.101	3.222	0.0013
Substance Use	0.155	4.991	0.0000
Behavioral Disengagement	0.285	9.475	0.0000
Denial	0.195	6.350	0.0000

## Abbreviations

TMD	temporomandibular disorders
AB	awake bruxism
SB	sleep bruxism
TMJ	temporomandibular joint
PSS-10	Perceived Stress Scale

## Appendix B

**Table A1:** Levels of statistical significance for post hoc pairwise comparisons of stress-coping strategies chosen in population presenting TMD symptoms.

Strategy	Planning	Active Coping	Self-Blaming	Seeking Instrumental Support	Seeking Emotional Support	Acceptance
Planning						
Active coping	chi^2^ = 0.674; *p* = 0.4110					
Self-Blaming	chi^2^ = 0.934; *p* = 0.3340	chi^2^ = 0.106; *p* = 0.7750				
Seeking instrumental Support	chi^2^ = 8.980; *p* = 0.0027	chi^2^ = 2.916; *p* = 0.0880	chi^2^ = 19.758; *p* = 0.000			
Seeking Emotional Support	chi^2^ = 12.025; *p* = 0.0005	chi^2^ = 4.752; *p* = 0.0293	chi^2^ = 6.277; *p* = 0.0122	chi^2^ = 0.223; *p* = 0.6370		
Acceptance	chi^2^ = 29.154; *p* = 0.0000	chi^2^ = 16.991; *p* = 0.0000	chi^2^ = 19.758; *p* = 0.0000	chi^2^ = 5.871; *p* = 0.0154	chi^2^ = 3.812; *p* = 0.0510	

**Table A2:** Levels of statistical significance for post hoc pairwise comparisons of stress-coping strategies chosen in population presenting awake bruxism symptoms.

Strategy	Planning	Active Coping	Self-Blaming	Seeking Instrumental Support	Seeking Emotional Support	Acceptance
Planning						
Active coping	chi^2^ = 0.005; *p* = 0.9430					
Self-Blaming	chi^2^ = 0.000; *p* = 1.0000	chi^2^ = 0.005; *p* = 0.9430				
Seeking instrumental Support	chi^2^ = 4.389; *p* = 0.0362	chi^2^ = 4.679; *p* = 0.0305	chi^2^ = 4.389; *p* = 0.0362			
Seeking Emotional Support	chi^2^ = 5.447; *p* = 0.0196	chi^2^ = 5.770; *p* = 0.0163	chi^2^ = 5.447; *p* = 0.0196	chi^2^ = 0.570; *p* = 0.4500		
Acceptance	chi^2^ = 16.385; *p* = 0.0000	chi^2^ = 16.934; *p* = 0.0000	16.385; *p* = 0.0000	chi^2^ = 3.874; *p* = 0.0490	chi^2^ = 2.997; *p* = 0.0830	

**Table A3:** Levels of statistical significance for post hoc pairwise comparisons of stress-coping strategies chosen in population presenting sleep bruxism symptoms.

Strategy	Planning	Active Coping	Self-Blaming	Seeking Instrumental Support	Seeking Emotional Support	Acceptance
Planning						
Active coping	chi^2^ = 0.544; *p* = 0.4610					
Self-Blaming	chi^2^ = 0.305; *p* = 0.5810	chi^2^ = 0.034; *p* = 0.8540				
Seeking instrumental Support	chi^2^ = 7.556; *p* = 0.0060	chi^2^ = 4.057; *p* = 0.0440	chi^2^ = 4.836; *p* = 0.0279			
Seeking Emotional Support	chi^2^ = 5.357; *p* = 0.0206	chi^2^ = 2.492; *p* = 0.1140	chi^2^ = 3.111; *p* = 0.0780	chi^2^ = 0.668; *p* = 0.4140		
Acceptance	chi^2^ = 24.828; *p* = 0.0000	chi^2^ = 18.113; *p* = 0.0000	chi^2^ = 19.705; *p* = 0.0000	chi^2^ = 3.873; *p* = 0.0490	chi^2^ = 2.997; *p* = 0.0830	
